# Analytical Validation and Capabilities of the Epic CTC Platform: Enrichment-Free Circulating Tumour Cell Detection and Characterization

**DOI:** 10.5772/60725

**Published:** 2015-05-05

**Authors:** Shannon L. Werner, Ryon P. Graf, Mark Landers, David T. Valenta, Matthew Schroeder, Stephanie B. Greene, Natalee Bales, Ryan Dittamore, Dena Marrinucci

**Affiliations:** 1 Epic Sciences, Inc., San Diego, CA, USA

**Keywords:** Analytical Validation, Biomarker, Circulating Tumour Cells, CTC, CTM, Clinical Feasibility, Epic CTC Platform, Fluid Biopsy, Liquid Biopsy, Metastasis

## Abstract

The Epic Platform was developed for the unbiased detection and molecular characterization of circulating tumour cells (CTCs). Here, we report assay performance data, including accuracy, linearity, specificity and intra/inter-assay precision of CTC enumeration in healthy donor (HD) blood samples spiked with varying concentrations of cancer cell line controls (CLCs). Additionally, we demonstrate clinical feasibility for CTC detection in a small cohort of metastatic castrate-resistant prostate cancer (mCRPC) patients. The Epic Platform demonstrated accuracy, linearity and sensitivity for the enumeration of all CLC concentrations tested. Furthermore, we established the precision between multiple operators and slide staining batches and assay specificity showing zero CTCs detected in 18 healthy donor samples. In a clinical feasibility study, at least one traditional CTC/mL (CK+, CD45-, and intact nuclei) was detected in 89 % of 44 mCRPC samples, whereas 100 % of samples had CTCs enumerated if additional CTC subpopulations (CK-/CD45- and CK+ apoptotic CTCs) were included in the analysis. In addition to presenting Epic Platform's performance with respect to CTC enumeration, we provide examples of its integrated downstream capabilities, including protein biomarker expression and downstream genomic analyses at single cell resolution.

## 1. Introduction

Over 90 % of cancer-related deaths from solid tumours are caused by complications of tumour metastasis [1]: the translocation of primary tumour cells to a distant tissue, followed by adaptation to and colonization of the microenvironment of a secondary site to facilitate tumour cell proliferation and the macroscopic formation of metastatic lesions [2, 3]. Circulating tumour cells (CTCs) are thought to represent the haematologic phase of tumour metastasis, as CTC detection and enumeration are greater in metastatic patients than those with high-risk or benign disease [4, 5].

CTCs were first discovered in the late 1800s [5, 6] and exist in frequencies in the range of one in one billion blood cells [2, 5]. Despite their rare nature, monitoring disease by the detection of CTCs has several key advantages over solid tissue biopsies [7]. CTCs are accessible via peripheral venous phlebotomy, which is less invasive to patients than solid tissue biopsies. In addition, some solid tissue biopsies require expensive radiographic imaging to guide biopsy needles, and can be potentially hazardous to patients who may already be weakened by current or previous history of cancer treatment. Importantly, there can also be significant intra-patient tumour evolution over time [8][Bibr bibr9-60725][Bibr bibr10-60725]–[11], for which blood collection can represent a real-time fluid biopsy, and is more amenable for repeat sampling than tissue biopsies.

The enumeration of CTCs has clinically validated prognostic value to predict progression free survival (PFS) and overall survival (OS) in metastatic breast cancer [12], prostate cancer [13] and colorectal carcinoma [14] patients using the CellSearch platform. Beyond enumeration, the molecular characterization of CTCs has potential to predict response to therapy [4, 15]. The integration of CTC enumeration and biomarker expression analysis has been proposed for use in early clinical development of therapeutics, as intermediate endpoints in clinical trials, and in stratification of patients for targeted therapy [16, 17].

CTC heterogeneity has been observed both within individual patients and across cohorts of patients, displaying a range of gene or protein expression signatures [4], cell size [18, 19], and cell density [9]. This fundamental inter- and intra-patient heterogeneity has made it challenging to define a standard CTC definition and reference range for the development of CTC detection platforms. Due to the rare nature of CTCs, several CTC detection and characterization methods utilize enrichment strategies to isolate CTCs from peripheral blood cells. Such enrichment techniques rely on CTC expression of epithelial markers (EpCAM, cytokeratin), depletion of cells expressing a common leukocyte marker (CD45), selection of cells with specific physical properties (cell size, density, deformity), or a combination of epitope and physical property selection [16, 17].

Positive selection of CTCs is the most common mechanism of CTC enrichment and is utilized by many technologies in development, including CellSearch, the only platform currently FDA-cleared for prognostic applications [5, 16]. However, emerging literature suggests that CTCs display degrees of epithelial epitope plasticity, and have been reported to have more than 10 times less EpCAM expression per cell than solid primary and metastatic tissue samples [20]. Additionally, common epithelial cell surface markers (EpCAM, E-Cadherin, cytokeratins) are often downregulated or absent in pluripotent cancer stem cells or cells undergoing epithelial-to-mesenchymal transition (EMT) [21, 22]. In preclinical models, cells undergoing de-differentiation or EMT have been associated with increased motility, invasiveness and tumour aggressiveness [23][Bibr bibr24-60725]–[25]. Thus, detection strategies that rely on epithelial marker enrichment might miss biologically relevant CTC subpopulations and hinder comprehensive analysis of CTC heterogeneity. To address this issue, some CTC detection platforms integrate negative selection, the depletion of CD45(+) cells from whole blood, as a method to enrich CTCs in an effort to detect epithelial marker-negative cells, and studies characterizing this strategy are ongoing [26][Bibr bibr27-60725]–[28].

Alternatively, size exclusion methods select for cells that are larger than white blood cells and are not biased by cell surface marker expression [16, 29–31]. However, studies of prostate cancer, breast cancer, and melanoma CTCs have found considerable overlap between the lower limit of CTC diameter and median white blood cell diameter [18, 26], [32], which impacts the ability of size exclusion methods to detect small CTCs. Size exclusion and micro-filtration systems can also reduce high CTC recovery due to cell membrane stress, thus reducing dynamic range and cell viability during the cell capture process [33], and some membrane filtration systems have been reported to show CTC capture variability, as well as frequent sample clogging on filters [34].

Many emerging CTC detection modalities make use of microfluidics to select via size and deformity [35], aid enrichment by increasing epitope availability [32, 36], [37], or assist in immunomagnetic positive or negative epitope selection [26]. Analogous to size exclusion and epitope selection, microfluidic techniques must utilize assumptions about the physical nature of CTCs they are engineered to detect. Without an established reference range or universal definition of CTC, any chosen parameters of CTC enrichment might bias sampling and miss biologically relevant CTCs. While microfluidic devices have displayed improved sensitivity of CTC detection and higher separation efficiency compared to first-generation approaches [33, 38], one drawback is the potential for clogging [38, 39], which has important implications for accurate CTC enumeration for patients with high CTC burden, or for the detection of CTC clusters. Additional drawbacks can include low sample throughput due to the complex integration of external electrical/magnetic fields, and prolonged processing time due to the device's high fluidic resistance [34, 39]. Additionally, CTC isolation using microfluidic chips typically requires a fresh blood sample to be processed within hours of patient blood draw at the clinical site, rather than allowing for shipment and blood sample processing at a centralized CLIA laboratory [26]. Importantly, processing an entire fresh blood sample through a microfluidic chip may preclude the ability to store morphologically intact CTCs in a biorepository for retrospective biomarker analyses.

To overcome the challenges outlined above, we developed an unbiased method to detect and characterize CTCs without cell enrichment, depletion or microfluidic manipulation, and with the added feature of being able to store samples in a central biorepository. While detection of CTCs has prognostic value for patient survival [5, 12–14], it is a comprehensive portrait of biomarker expression, heterogeneity and clonal evolution that has been proposed as having great promise to derive clinically actionable CTC signatures for drug development, patient stratification and evaluation of drug resistance mechanisms [16, 17], [40]. To this end, the Epic CTC Platform was designed with integrated downstream capabilities for the evaluation of protein (immunofluorescence) and genetic (FISH, NGS) biomarkers with single cell resolution.

The following report describes the analytical performance of the Epic CTC Platform, clinical feasibility for the detection of both traditional and non-traditional CTCs in mCRPC patients, and a description of the platform's downstream biomarker analytic capabilities.

## 2. Methods

### 2.1 Sample Receipt, Processing and CTC Enumeration

**Figure 1.**
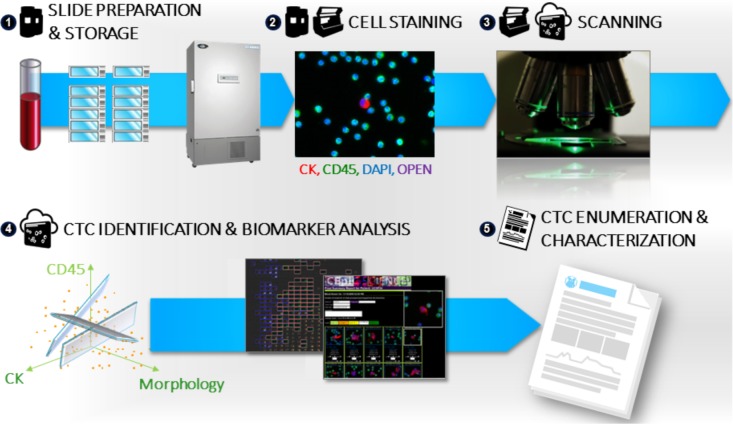
**Epic Platform workflow for sample preparation, CTC enumeration and biomarker analysis.** Upon patient blood sample receipt at Epic Sciences, 1) whole blood is lysed and nucleated cells (3 × 10^6^ per slide) are deposited onto 10-12 microscope slides and are frozen at −80 °C until analysis. 2) Two slides per patient sample are thawed and stained with a cocktail of antibodies including cytokeratin, CD45, DAPI to perform CTC enumeration, and a fourth fluorescent channel is available for the evaluation of protein biomarker expression. 3) Stained slides are scanned and 4) the resulting images are analysed using a multi-parametric digital pathology algorithm to detect CTC candidates and quantitate biomarker expression levels. CTC classifications are displayed in a web-based report and are confirmed by trained technicians. 5) CTC enumeration and biomarker expression results are compiled and reported.

**Figure 2.**
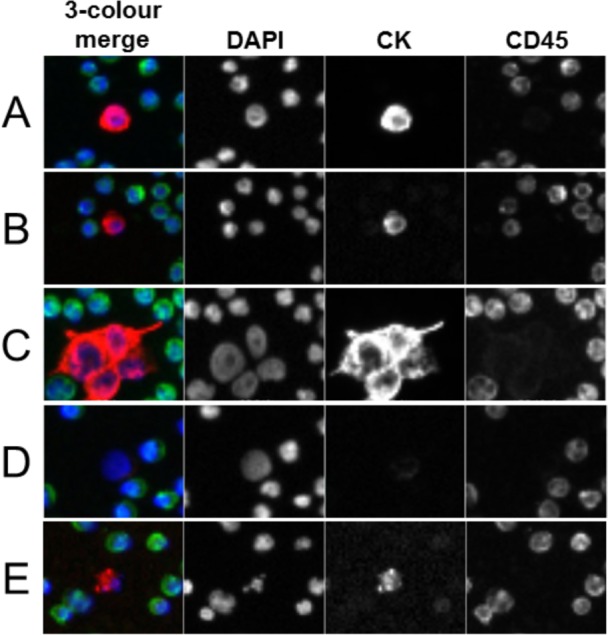
**Representative CTC subtypes detected by the Epic Platform.** CTCs from prostate cancer patient samples were enumerated using the Epic Platform. Representative 10X immunofluorescence images for the DAPI (blue), cytokeratin (red), CD45 (green) channels are shown for CTC subtypes and the surrounding white blood cells (WBCs), with the three-channel merge to the far left of each image. Classified CTC subtypes include **A)** Traditional CTCs (CK+, CD45-, DAPI+/intact), **B)** Small CTCs (CK+, CD45-, DAPI+/intact, with similar nuclear size to that of the surrounding WBCs), **C)** CTC clusters (two or more adjacent traditional CTCs that share cytoplasmic boundaries), **D)** CK- CTCs (CK-, CD45-, DAPI+/intact), and **E)** Apoptotic CTCs (CK+, CD45-, with DAPI staining pattern consistent with chromosomal condensation and/or nuclear fragmentation).

Samples were processed and analysed using the Epic CTC platform (Figure 1) as previously described [41][Bibr bibr42-60725]–[43]. Briefly, blood samples were collected in 10 mL cell-free preservative blood tubes (Streck, Omaha, Nebraska) and shipped to Epic Sciences. Red blood cell (RBC) lysis was performed using ammonium chloride solution. Following centrifugation, all nucleated cells were deposited on up to 12 glass slides per sample at a concentration of 3 × 10^6^ cells/slide and frozen at −80 °C until analysis. After thawing, two slides per sample were immunofluorescently (IF) stained with a cocktail of antibodies targeting cytokeratins (CK), CD45, and 4‘, 6-Diamidino-2-phenylindole, dihydrochloride (DAPI). Slides were scanned by Epic's rapid fluorescent scanning method [43], which analyses each nucleated cell per slide using a proprietary algorithm developed within the context of haemato-pathology standards. In short, the algorithm utilizes 90 cellular parameters, including marker expression and cell morphology, to differentiate candidate CTCs from surrounding white blood cells (WBCs) [43]. Candidate CTCs were identified and displayed in a web-based report, and trained technicians confirmed CTC candidates as being classified into one of the following categories (Figure 2):

**Traditional CTCs (Figure 2A)**: defined as cells CK(+), CD45(−), intact DAPI, and are generally larger and morphologically distinct from surrounding WBCs.

**Small CTCs (Figure 2B)**: defined as CK(+), CD45(−), intact DAPI cells that are the same size or smaller than the size of neighbouring WBCs.

**CTC Clusters (Figure 2C)**: defined as two or more adjacent CTCs, containing at least one traditional CTC, with shared cytoplasmic boundaries.

**CK(−) CTCs (Figure 2D)**: defined as CK(−), CD45(−), with DAPI intact.

**Apoptotic CTCs (Figure 2E)**: defined as CK(+), CD45(−) with a DAPI pattern of chromosomal condensation and/or nuclear fragmentation/blebbing that is consistent with the classic definition of apoptosis [44].

### 2.2 Analytical Validation of CTC Detection

**Figure 3.**
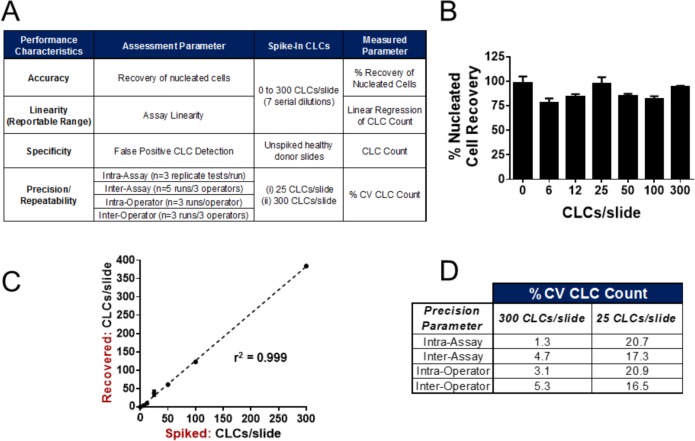
**Analytical Validation of the Epic Platform. A)** The analytical characteristics assessed to benchmark the performance of the Epic CTC platform. Varying concentrations of COLO-205 cell line cells (CLCs) were spiked into healthy donor blood, red blood cells lysed, and 3 × 10^6^ nucleated cells were deposited onto slides, ranging from 0-300 CLCs/slide. Slides were stained with a cocktail of CK, CD45 and DAPI antibodies, and assay accuracy, linearity, specificity and precision were determined as described in the methods. For each analysis, a “run” is comprised of three tests, with each test consisting of two replicate slides. **B)** The accuracy and repeatability of cell deposition was assessed calculating percent nucleated cell recovery (y-axis; Mean ± SEM) for one run each of six serial CLC dilutions (six, 12, 25, 50, 100 and 300 CLCs/slide), and for five slides of unspiked healthy donor (HD) blood (zero CLCs/slide). **C)** Assay linearity was characterized by plotting the actual CLCs/slide recovered (y-axis) versus the theoretical number of CLCs/slide (x-axis) for seven CLC concentrations (six slides tested/concentration), and the linear regression was calculated. Assay specificity was determined by measuring the number of CLCs detected on the unspiked healthy donor slides (zero CLCs/mL). **D)** Assay precision/repeatability was measured by calculating the percent coefficient of variation (%CV) for CLC counts from the 25 CLCs/slide and 300 CLCs/slide dilutions. Intra-assay variability was measured for one operator who performed one assay run, whereas inter-assay variability was measured across three operators who performed five assay runs total (one assay run per day). Intra-operator repeatability was measured for one operator who performed three assay runs on separate days, whereas inter-operator repeatability was measured for thee operators who performed one assay run each.

Cultured cancer cell line cells (CLCs; COLO-205) were spiked into whole blood specimens from healthy donors (HD) at varying concentrations ranging from six-300 CLCs/slide (six slides each of six, 12, 25, 50, 100, 300 CLCs/slide, and 24 additional slides were created for the 25 and 300 CLCs/slide dilution). Additionally, five slides of unspiked HD samples were prepared. All blood samples (spiked or unspiked) were processed as described above, and 3 × 10^6^ nucleated cells were deposited per slide. Slides were stained using the Epic standard three-colour assay (CK/CD45/DAPI), and scanned using the Epic CTC platform. One assay (staining/scanning) run consisted of three replicate tests of two slides per test (six slides total). Following scanning and CTC classification, the number of CLCs enumerated on two replicate slides was converted to CLCs per millilitre of blood, and the resulting values were used to assess four critical assay performance characteristics (Figure 3A):

- Cell deposition repeatability and accuracy
- Linearity
- Specificity
- Precision/repeatability

Cell deposition repeatability and assay accuracy were evaluated by measuring the DAPI counts per slide and calculating percent nucleated cell recovery for three replicate tests (two slides per test; six slides total) for each of six serial CLC dilutions (six, 12, 25, 50, 100 and 300 CLCs/slide), and for five slides of unspiked healthy donor (HD) blood (zero CLCs/slide). Percent coefficient of variation (%CV) of DAPI counts was calculated for each CLC dilution tested.

**Figure 4.**
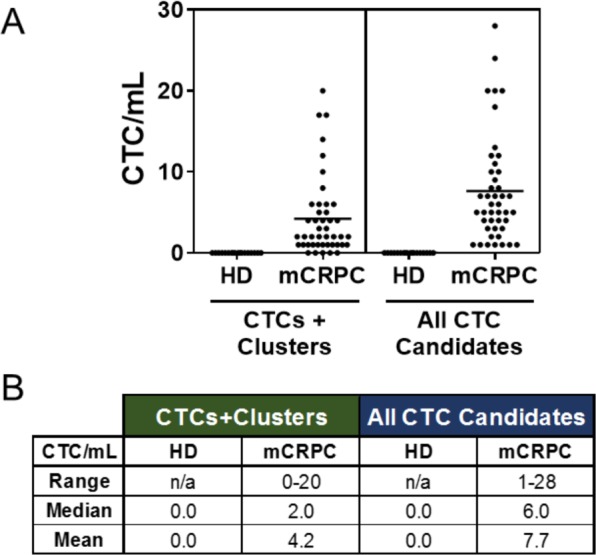
**Clinical feasibility of CTC enumeration in metastatic castrate-resistant prostate cancer patient samples.** Forty-four (44) mCRPC patient blood samples were tested for CTC enumeration using the Epic Platform, and the results were compared to those from 18 healthy donor (HD) blood samples. **A)** CTC incidence was calculated as CTC per millilitre (CTC/mL) of patient blood for traditional CTCs and CTC clusters (left, CTCs + clusters) and all CTC candidates (right; CTCs, CTC clusters, CK- CTCs, and apoptotic CTCs). Each dot on the graph is representative of the CTC/mL value for that patient sample. **B)** Summary of the range, median and mean CTC/mL values for 18 healthy donor and 44 mCRPC samples for traditional CTCs and CTC clusters (left) and all CTC candidates (right).

Assay linearity was evaluated by plotting the actual CLCs/slide recovered versus the theoretical number of CLCs/slide for each of the CLC concentrations tested, where each concentration was tested in triplicate tests (two slides per test). The linear regression was calculated. Assay specificity was determined by measuring the number of CLCs detected on the five unspiked healthy donor slides (zero CLCs/mL), as well as in 18 healthy donor samples (two slides tested per sample) in a clinical feasibility analysis (Figure 4).

Assay precision/repeatability was measured by calculating the percent coefficient of variation (%CV) of CLC counts from the 25 CLCs/slide and 300 CLCs/slide dilutions. Intra-assay variability was measured for one operator who performed three tests on one day (two replicate slides/test; six slides total), whereas inter-assay variability was measured across three operators who performed five assay runs total (one run per day), with each run consisting of three tests of two slides per test (30 slides total). Intra-operator repeatability was measured for one operator who performed three assay runs (one run per day), with each run consisting of three replicate tests of two slides/test (18 slides total). Inter-operator repeatability was measured for three operators who performed one assay run each (18 slides total).

### 2.3 Clinical Feasibility of CTC Detection

Forty-four (44) blood samples from all-comer metastatic castrate-resistant prostate cancer (mCRPC) patients were sent to Epic Sciences, processed onto slides, and two slides per sample were tested for CTC enumeration as described above. CTC enumeration (CTC/mL) was compared to that found for 18 healthy donor blood samples (two slides tested per sample), which also further addressed assay specificity. The prevalence of CTC clusters, CK- CTCs and apoptotic CTCs are also reported for the mCRPC cohort as percent of samples containing each subtype.

## 3. Results

### 3.1 Analytical Validation

To assess assay accuracy, linearity, specificity and precision/repeatability, the CK(+), CD45(−), DAPI(+) COLO-205 cancer cell line control (CLC) was spiked into healthy donor blood and processed onto slides as mock clinical samples. Six (6) slides each of six CLC dilutions (six, 12, 25, 50, 100, 300 CLCs/3 × 10^6^ WBCs per slide and 24 additional slides for both the 25 and 300 CLCs/slide dilution) were prepared, as well as five slides of unspiked HD samples. A description of the assay performance characteristics evaluated is described in Figure 3A.

#### 3.1.1 Cell Deposition Repeatability, Assay Accuracy and Linearity

To evaluate the repeatability of the cell deposition onto slides, one operator performed three replicate CTC enumeration tests (two slides/test; six slides total) for each of the six CLC dilutions, as well as for five replicate unspiked HD slides, for a total of 41 slides. The numbers of nucleated cells per slide (DAPI counts) were counted, and the percent recovery of nucleated cells per CLC dilution was calculated (Figure 3B). Across 41 slides, the mean percent recovery was 88 % (2.64×10^6^ nucleated cells/slide), with an observed coefficient of variation (%CV) of 9.7 %. The %CVs for each cell dilution were 6.7 % (zero CLCs/slide), 6.3 % (six CLCs/slide), 3.0 % (12 CLCs/slide), 7.0 % (25 CLCs/slide), 2.7 % (50 CLCs/slide), 3.8 % (100 CLCs/slide), and 1.2 % (300 CLCs/slide). Assay linearity was evaluated by plotting the number of CLCs recovered per slide for each of the spike-in concentrations from three replicate tests (two slides/test; six slides total) against the calculated CLC spike-in concentration (Figure 3C), and the linear regression was calculated. The assay was shown to be linear across all sample dilutions tested (r^2^=0.999). Importantly, zero CTCs were enumerated in the unspiked healthy donor slides, thereby showing assay specificity. Assay specificity is further established in the clinical feasibility testing of 18 healthy donor samples (two slides per sample tested), as shown in Figure 4.

#### 3.1.2 Assay Precision and Repeatability

Precision of the Epic Platform was assessed intra- and inter-assay as well as intra- and inter-operator by calculating the observed variation in the enumeration of CLCs in the 25 CLCs/slide and 300 CLCs/slide dilutions (Figure 3D). One assay run consisted of three replicate tests, with two slides stained per test (six slides total) per CLC concentration. Intra-assay repeatability was assessed by calculating the %CV of CLCs detected per millilitre (CLCs/mL) of blood from slides prepared by a single operator who performed one assay run (six slides total) for both the 25 CLCs/slide and 300 CLCs/slide dilutions. The observed %CVs were calculated to be 20.7 % and 1.3 %, respectively. Inter-assay repeatability was determined from calculating the %CV of CLCs/mL blood detected based upon five assay runs performed by three operators on separate days, for a total of 30 slides evaluated per CLC dilution. The resulting %CVs calculated were 4.7 % for 300 CLCs/slide, and 17.3 % for 25 CLCs/slide. Intra-operator precision was evaluated for one operator who performed one assay run on three separate days (18 slides total), which resulted in %CVs of 3.1 % for 300 CLCs/slide and 20.9 % for 25 CLCs/slide. Finally, inter-operator precision was assessed for three operators who performed one assay run each (18 slides total) on separate days. For this parameter, the observed %CV for the 300 CLCs/slide dilution was found to be 5.3 % and 16.5 % for the 25 CLCs/slide dilution.

Coefficient of variation for the detection of 300 CLCs/slide increased from 1.3 % for samples analysed by a single operator in a single run to only 3.1 % when the single operator performed a single run on three separate days, whereas it increased to only 4.7 % when three operators performed five runs in total over five days. For the 25 CLCs/slide dilution, the %CV in CLC detection was the same if an operator performed a single run on one day, or if he performed one run on three separate days (20.7 % and 20.9 %, respectively). Similarly, the %CV in detection of 25 CLCs/slide was found to be similar between a single operator who performed one run and multiple operators who performed runs on separate days. The variability for the detection of 25 CLCs/slide was found overall to be higher than that for 300 CLCs/slide, which is not unexpected due to the inherent variability of performing serial cell dilutions, and the %CV was found to be at or under 20 % for all parameters tested.

### 3.2 Clinical Feasibility

To establish clinical feasibility, we tested 44 all-comer metastatic castrate-resistant prostate cancer (mCRPC) samples, and compared CTC enumeration to that from 18 healthy donor samples (Figure 4). Traditional CTCs (CK+/ CD45-/intact nuclei) and clusters of traditional CTCs were detected in 89 % of the mCRPC samples analysed with a range of 0-20 CTC/mL (median=2.0 CTC/mL), whereas 23 % of samples were found to have CTC clusters. Considering all possible CTC candidates (including non-traditional CK(−) CTCs and apoptotic CTCs), 100 % of the mCRPC samples had detectable CTCs (Figure 4B) with a range of 1-28 CTC/mL (median=6.0 CTC/mL). In this cohort, we observed that 70 % of samples had CK(−) CTCs, whereas 77 % had apoptotic CTCs. In contrast, zero CTCs were enumerated in the 18 healthy donor samples tested, which further exhibits the specificity of the Epic Platform. So far, CTC and biomarker stability on patient slides have been demonstrated for up to one year, with studies ongoing to determine longer term storage stability (data not shown).

### 3.3 Downstream Capabilities of the Epic Platform

In addition to CTC enumeration, the Epic CTC Platform was designed with integrated downstream capabilities for the evaluation of cell morphology characteristics, protein biomarker expression and genomic analyses (FISH and NGS). The platform has an open fourth fluorescent channel for the evaluation of protein biomarker expression in patient CTCs (Figure 1, Step 2 of the CTC Assay workflow), with a fifth channel currently in development. Currently, a wide variety of fourth channel markers have been developed targeting multiple disease indications, EMT cell markers and drug sensitivities. Using the Epic Platform, it is possible to simultaneously evaluate the expression of targetable protein biomarkers (IF), the presence of specific driver genomic alterations (FISH) and genome-wide copy number alterations (NGS) from a single tube of blood, and importantly, within individual patient CTCs. Both FISH and NGS analysis can be performed on single CTCs detected using the immunofluorescence assay workflow (Figure 1). The potential to integrate the analysis of multiple biomarkers from a single patient blood sample, including genomic, protein and morphological endpoints, holds great promise for better understanding of disease progression, heterogeneity and sensitivity/resistance to targeted therapies [16, 40], [45].

**Figure 5.**
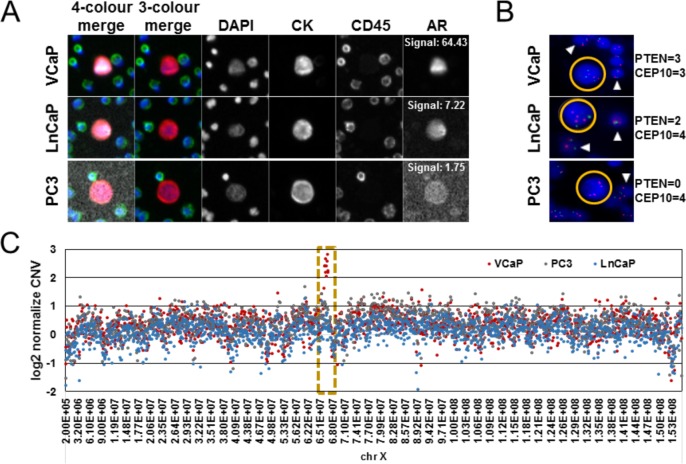
**Epic Platform capabilities for the evaluation of protein and genetic biomarkers.** Human prostate cancer cell line control cells (CLCs; VCaP, LnCaP or PC3) were spiked into healthy donor blood, processed onto slides and stained with CK, CD45, DAPI and N-terminal androgen receptor (AR) antibodies. Additional slides were processed for *PTEN* loss by FISH. Subsequently, individual CLCs were recovered and analysed for whole genome copy number variation by NGS. **A)** Representative images (10X) of individual CLCs detected, each with varying levels of AR expression (AR signal denoted in white). **B)** Representative images of *PTEN* gene deletion status in CLCs (yellow circles) and surrounding WBCs (white carrots), as determined by PTEN FISH analysis. Blue: DAPI, Red: CEP10 signals, Green: PTEN signals. The number of PTEN and CEP10 signals found in each CLC example are reported to the right of the image. **C)** Comparison of log2 CNV (y-axis) found within isolated VCaP (red), PC3 (grey), and LnCaP (blue) CLCs across the X chromosome (x-axis). Each data point represents the relative copy number within a 100,000 bp window normalized to healthy donor control WBC CNV. The highlighted window (yellow dotted line) contains the *AR* gene.

As proof of concept (Figure 5), slides were created from healthy donor blood samples spiked with prostate cancer cell lines (VCaP, LnCaP and PC3), and were analysed to confirm the presence of known protein and genetic markers associated with prostate cancer disease progression and/or resistance to targeted therapies. Common molecular alterations in mCRPC include changes in androgen receptor (AR) signalling through alterations in AR protein expression levels and gene copy number variations, the presence of *AR* splice variants and mutations, and altered PI3K-axis signalling through *PTEN* gene deletions 46–49]. Relative AR protein expression was analysed in VCaP, LnCaP and PC3 CLCs detected using the fourth channel capability of the Epic Platform (Figure 5A), and confirmed with the relative AR expression known to be found in the high-, medium- and low-AR expressing cell lines, respectively. *PTEN* tumour suppressor gene loss was evaluated in these prostate cancer CLCs by FISH (Figure 5B), where the signals for PTEN (green) and CEP10 (red) can be compared between the CLC (yellow circle) and the surrounding white blood cells (white carrots). In all three CLC examples, the CEP10 signals were found to be greater than two, which is indicative of polyploidy and is consistent with the tumour origin of the CLCs. The VCaP CLC was shown to have *PTEN* non-deleted status (PTEN=CEP10; three signals each), whereas LnCaP showed heterozygous *PTEN* loss (PTEN=2; CEP10=4) and PC3 showed homozygous *PTEN* loss (PTEN=0, CEP10=4). The presence of surrounding WBCs provide ample controls for evaluating the false-positive rate of detection of genetic alterations. Additionally, *AR* gene copy number variation (CNV) was evaluated in single CLCs using next-generation sequencing (NGS; Figure 5C). As reported previously, *AR* gene amplification was found in the VCaP CLC [50, 51], but not the LnCaP or PC3 CLCs. Using the platform's integrated downstream analysis, multiplexed evaluation of genomic and protein biomarkers within a patient sample and individual patient CTCs offers a unique opportunity to better understand mechanisms of resistance to therapy and to inform the optimization of targeted therapy regimens.

As recently summarized by Macaulay and Voet, substantial advances in single cell genomic analyses for the detection of point mutations, copy number variation (CNV), loss of heterozygosity (LOH) and structural variants have been made [52]. However, amplifying DNA material via whole genome amplification (WGA) has the inherent risk of creating bias and false-positive/negative results. An alternative strategy to increase DNA quantity while preventing the complications associated with single cell analysis, would be to pool individual CTCs isolated from a patient sample by phenotypic subtype. However, this would negatively impact the ability to evaluate intra-patient heterogeneity. Development of quality control (QC) criteria for evaluating the quality of both the NGS library and post-sequencing data has been described and implemented to avoid such false-positive and false-negative results [53]. Additionally, secondary validation studies utilizing immunofluorescence to identify CTCs, followed by DNA FISH may confirm the incidence of specific genomic aberrations in patient CTCs, for which the Epic Platform is suitable.

## 4. Discussion

This study encompasses the analytical validation of the Epic CTC detection platform where we assessed critical assay performance characteristics including assay accuracy, linearity, specificity and precision/repeatability (intra-assay, inter-assay, intra-operator and inter-operator). Notably, the platform demonstrated a high percentage of nucleated cell recovery for all CLC concentrations tested (Figure 3B), and showed excellent assay linearity (r^2^ = 0.999) (Figure 3C). Furthermore, the assay is highly repeatable for detecting CLCs at multiple dilutions within and across assay runs and multiple operators (Figure 3D). The assay is also highly specific, in that zero CTCs were detected in unspiked healthy donor samples (Figure 3C, Figure 4). While in-depth clinical feasibility studies are underway, data from a small cohort of clinically confirmed all-comer mCRPC patient samples tested with the Epic CTC Platform showed that ≥1 traditional CTC/mL was detected in 89 % of patient samples, whereas 100 % of samples had ≥1 CTC/mL when additionally considering the CK(−) and apoptotic CTC subpopulations. In this patient cohort, we observed the presence of multiple CTC subtypes, including CK(+) CTCs, CTC clusters, CK(−) CTCs and apoptotic CTCs. This is in contrast to the healthy donor samples tested, in which zero CTCs were enumerated in all 18 samples. In Figure 5, we discuss the downstream capabilities of the Epic Platform, including methods to evaluate protein (immunofluorescence) and genetic (FISH, NGS) biomarkers.

In the last 10 years, great strides have been made with the technological development of CTC detection strategies, as well as advancements in the understanding of CTC biology, resulting in over 16,000 publications. However, further investigation of CTC utility in directing personalized medicine is warranted, and many studies and trials are now underway to address this. Low CTC abundance poses a challenge with respect to having a statistically significant number of cells for biomarker analyses, and thus to the evaluation of tumour heterogeneity. Low CTC incidence has been reported for non-metastatic or locally advanced prostate [32, 54] or locally advanced pancreatic adenocarcinoma [55], and non-metastatic colorectal cancer [56], which may limit the utility of CTC assays for diagnosis of early stage disease. Increasing the sensitivity of CTC detection may address this issue; however, increased sensitivity may result in the detection of false-positive results in healthy controls or in patients with benign disease [57]. Additionally, some cancer indications such as ovarian [58] and NSCLC [59] have reportedly low CTC numbers; however, the low abundance of CTCs identified may be a result of the CTC detection platform selected and reliance on using CTC enrichment strategies. Emerging data show that CTCs are detected in larger numbers in these indications when using epithelial marker-independent approaches [60][Bibr bibr61-60725]–[62].

Detection of CTCs with an epithelial-to-mesenchymal transition (EMT) phenotype remains a challenge, and their functional and clinical relevance are still under investigation. CTC platforms that rely on epithelial marker enrichment are ill-suited to detect these cells [20]; however, there is no universal biomarker that ensures the detection of mesenchymal CTCs. For example, some EMT markers (i.e., vimentin) are expressed on the surrounding leukocytes, and CTCs show a broad range of phenotypes during EMT and may express neither cytokeratins nor EMT markers [4]. In addition to identifying additional mesenchymal markers, it is unclear as to whether mesenchymal CTCs are even capable of seeding distal metastases, as these cells may be unable to undergo the reverse “mesenchymal-to-epithelial” transition [63]. CTCs may have an intermediate phenotype where they partially downregulate epithelial markers while partially upregulating mesenchymal markers, or express neither. These highly plastic cells suggest their stemness [64] but additional studies are warranted to evaluate their clinical significance.

The Epic CTC Platform uses an unbiased CTC detection approach in which additional CTC subpopulations including CTC clusters, CK(−) CTCs, small CTCs and apoptotic CTCs are enumerated. While the biological significance of each CTC subtype is still under investigation, emerging literature suggests that each subtype may play an important role in tumour metastasis, epithelial-to-mesenchymal transition (EMT), and as potential markers for cancer therapeutic responses. Although not extensively validated as a biomarker, the presence of CTC clusters is reported to be a negative prognostic factor for survival in cohorts of small-cell lung cancer [65], as well as breast cancer and prostate cancer patients [66]. Preclinical models suggest that CTC clusters might have survival advantages in blood circulation including resistance to programmed cell death and physical sheer forces [65][Bibr bibr66-60725][Bibr bibr67-60725]–[68]. There is limited published data on the biological relevance of CK(−) CTCs; however, it has been shown that CTCs of epithelial origin can display a range of both epithelial and non-epithelial gene biomarker signatures [4, 32, 69, 70]. Similarly, in-depth literature describing the biological significance of apoptotic CTCs is limited, although the enumeration of apoptotic CTCs has been explored as a potential drug response biomarker [71]. Characterization of the prevalence and functional significance of these CTC subtypes as clinical markers for predicting sensitivity to targeted therapies and understanding disease progression is of great interest, and is currently the subject of multiple ongoing clinical studies at Epic Sciences.

An important feature of the Epic Platform is the capacity for the creation of a biorepository of patient slides, which may be frozen and stored for retrospective biomarker analyses. While one CTC test uses two slides, approximately 10-12 slides are created per patient sample and the remaining slides are banked at −80 °C. In the wake of new biomarker discoveries and/or generation of biomarker hypotheses for a particular indication, banked patient slides may be evaluated retrospectively and correlated with clinical outcome data. So far, we have established patient slide stability for storage at −80 °C for up to one year with respect to their ability to retain CTCs and biomarker expression. Longer-term stability studies of frozen patient slides are currently underway.

While advancements in genomics and proteomics have contributed to our molecular understanding of cancer, emerging research also highlights inter-patient [72] as well as spatial [73][Bibr bibr74-60725][Bibr bibr75-60725][Bibr bibr76-60725]–[77] and temporal [8][Bibr bibr9-60725][Bibr bibr10-60725]–[11] intra-patient heterogeneity, both of which limit the efficacy of targeted therapies and thus compromise patient outcomes. Characterizing intra-patient heterogeneity has been posited as a means to intelligently guide precision-targeted therapies based on the current state of disease [7, 78], [79] and the development of resistance mechanisms to therapy. A comprehensive approach based on the detection of a panel of protein and genetic CTC biomarkers can give rise to a patient tumour's molecular signature, which will not only inform the development of targeted therapeutic strategies, but will also allow for patient surveillance based upon molecular alterations over time. Evaluation of biomarker panels in a liquid biopsy may provide the molecular landscape of primary and metastatic lesions, while offering the opportunity to evaluate molecular tumour evolution as a real-time film instead of a single frame snapshot.

## 5. Conflict of Interest

Authors SLW, RPG, ML, DTV, MS, SG, NB, RD and DM are employees of Epic Sciences, Inc.

## 6. Compliance with Ethical Research Standards

All patients included in this study gave informed consent for blood collection and CTC analysis.
